# Berberine Protects Mice Against Dextran Sulfate Sodium-Induced Colitis by Activating mTORC1 Pathway

**DOI:** 10.3389/fphar.2019.00786

**Published:** 2019-07-11

**Authors:** Qingjun Li, Xinyan Qu, Xiaogang Pang, Yue Song, Liyuan Chen, Qiuyue Xiao, Linlin Sun, Xiaolong Wang, Huimin Zhang, Dongmei Qi, Zhenguo Wang

**Affiliations:** ^1^Experimental Center, Shandong University of Traditional Chinese Medicine, Jinan, China; ^2^Key Laboratory of Basic Research of Traditional Chinese Medicine in Shandong Province, Shandong University of Traditional Chinese Medicine, Jinan, China; ^3^Laboratory of Immunology for Environment and Health, Shandong Analysis and Test Center, Qilu University of Technology (Shandong Academy of Sciences), Jinan, China; ^4^College of Pharmacy, Shandong University of Traditional Chinese Medicine, Jinan, China; ^5^Shandong Academy of Chinese Medicine, Jinan, China; ^6^School of Information Management, Shandong University of Traditional Chinese Medicine, Jinan, China

**Keywords:** berberine, ulcerative colitis, metabolism of amino acid, mTORC1, Treg cells

## Abstract

Berberine is a plant alkaloid that can be extracted from many Chinese herbs. It has been reported that berberine could protect mice from ulcerative colitis, but the mechanism remains unclear. The current study’s aim was to determine the potential mechanism by which berberine exhibits its anti-inflammatory function. Mice with colitis induced by dextran sulfate sodium (DSS) were administered with berberine at 50 mg/kg by gavage. Berberine significantly increased the proportion of regulatory T cells (Treg cells). The targeted metabolomics analysis was then performed to find that glutamine and glutamate metabolism played an important role in the process of regulating immune response. mTORC1 pathway was reported to closely relate with glutamine metabolism. As a result, the relative expression levels of downstream effector genes of mTORC were further determined, and data obtained showed that berberine could significantly increase the relative expression levels of S6K1 and 4EBP1. In addition, rapamycin was used to inhibit mTORC1 signaling, and it was found that colon length, disease associated index (DAI), and proportion of Treg cells of mice in the rapamycin-DSS group were not different from those of mice in the rapamycin/berberine-DSS group. Together, these results suggest that berberine exhibits significant protective effects against DSS colitis by activating the mTORC1 pathway to increase the proportion of Treg cells.

## Introduction

Ulcerative colitis (UC), one type of inflammatory bowel disease, is a chronic inflammatory disease featured with relapse and remission of mucosal inflammation. The incidence of UC is rising worldwide especially in developed countries. The pathogenesis is related with multiple factors such as genetic predisposition, environmental risk factors, medications, and intestinal immune imbalance. The main drugs used to treat UC are aminosalicylates, glucocorticoids, and immunosuppressive agents (Shah and Feller, 2009; Danese and Fiocchi, 2011; Ungaro et al., 2017). However, there are still patients who do not respond to the drugs. The cost of treatment is a great challenge for patients and governmental health care. Novel treatment should be important for UC.

Chemical compounds extracted from plants have been proved to exhibit many bioactivities including anti-inflammation, antioxidant, and anti-virus activities (Su and Hsieh, 2011; Lin et al., 2015; Bao et al., 2016; Law et al., 2017). The compounds characterized with anti-inflammatory effects provide an opportunity for treatment of UC. Berberine is a plant alkaloid that can be extracted from many Chinese herbs such as *Rhizoma coptidis*, *Cortex phellode*, and* Berberis* (Zhou et al., 2013). Berberine has been documented to possess many beneficial biological effects: berberine has been reported to protect mice from DSS colitis (Li et al., 2016; Yu et al., 2018); berberine exerts an anti-apoptosis effect against cytomegalovirus through its antioxidative properties (Zhuang et al., 2018); and it has been established that berberine plays a role in regulating lipid metabolism, organismal energy balance, and diabetes (Wang et al., 2014; Zhang et al., 2014; Wang et al., 2017; Sun et al., 2018).

With regard to protective effects against DSS colitis, the mechanism of berberine remains unclear and needs further analysis. It has been reported that berberine could regulate Treg/Th17 balance to treat DSS-induced colitis, and the mechanistic target of rapamycin complex 1 (mTORC1) pathway takes an important role in the Treg regulation and intestinal inflammation (Chapman and Chi, 2014; Brandt et al., 2018; Cui et al., 2018). Therefore, we proposed that berberine could treat colitis by regulating Treg cells *via* mTORC1 pathway. Targeted metabolomics provides an opportunity for the research of mechanisms of berberine, and LC MS/MS was used in this study to determine concentrations of 22 amino acids to analyze the mechanism of berberine (Qi et al., 2018).

In our present study, the protective effects of berberine against DSS colitis were evaluated by disease associated index (DAI), colon length, weight loss, and colonic tissue histological analysis. The amino acids in the colon were determined by LC MS/MS to analyze the mechanism by which berberine protects mice from DSS colitis.

## Materials and Methods

### Regent and Pharmacological Compounds

The standards of lysine, proline, valine, histidine, phenylalanine, glutamine, arginine, threonine, methionine, tryptophan, leucine, isoleucine, pyroglutamic acid, serine, asparagine, aspartic acid, γ-aminobutyric acid, glutamic acid, tryptophan, taurine, and alanine were obtained from Sigma-Aldrich. Methanol and acetonitrile for high performance liquid chromatography tandem mass spectrometry (HPLC-MS/MS) and methanol for chromatography were obtained from Merck. The dextran sulfate sodium (DSS, MW = 36–50 kDa) was a product of MP Bioscience, and berberine (purity > 95%) was purchased from the National Institutes for Food and Drug Control (Beijing, China). The rapamycin was bought from Sigma-Aldrich (lot number: 553211).

### Animals and Model

Female Balb/c mice (7–9 weeks) were purchased from the Beijing Vital River Laboratory Animal Technology Co., Ltd. The mice were housed under constant condition (24–25°C, 70–75% humidity) with 12-h light–dark cycles and fed with diet and water *ad libitum*. All the animal experiments protocols were reviewed and approved by the Animal Ethics Committee of the Shandong University of Traditional Chinese Medicine, and the animal experiments were conducted complying with the rules of the Shandong Administration Office of Laboratory Animals.

The mice were divided randomly into normal control (NC), 2% DSS-only, berberine (50 mg/kg, water), berberine (50 mg/kg, 2% DSS), rapamycin–2% DSS (Rapa-DSS), and rapamycin/berberine–2% DSS (Rapa/Berberine-DSS) groups. The mice in groups of DSS only, berberine-DSS, Rapa-DSS, and Rapa/Berberine-DSS were exposed to 2% DSS dissolved in drinking water for seven consecutive days. Berberine was dissolved in water and orally administered to mice, while mice in the normal control and 2% DSS-only groups were administered with the same volume of water. Rapamycin was injected daily intraperitoneally at dose of 1.5 mg/kg to inhibit the mTORC1 signaling. The start of treatment and the induction of colitis were both performed simultaneously. At the end of experiments, the colon length and weight loss were determined, and the DAI was assessed according to the method described by (Chen et al., 2017).

### Histological Evaluation

Colons were fixed in 4% neutral-buffered paraformaldehyde for at least 24 h. After being fixed, the colons were embedded in paraffin and processed for histological analysis. The colons were cut into 5-μm sections and stained with hematoxylin and eosin (H&E). The morphological changes were scored blindly according to the method described by Macia et al. (2015) as follows: no mucosal change scores 0; lymphoepithelial lesions score 1; surface mucosal damage scores 2; and extensive mucosal damage, lesions occur in deeper structure scores 3; inflammatory cell infiltration, occasional cell infiltration scores 0; increased infiltration of infiltrating cell scores 1; extension of infiltration to the submucosa scores 2; and transmural extension of the inflammatory cells scores 3.

### Antibodies

Antibodies against mouse CD3 (100216), CD8a (100744), and CD25 (102038) were purchased from BioLegend; Foxp3 (560401), B220 (552094), and CD4 (552775) were purchased from BD Biosciences.

### Flow Cytometry

Samples of spleen and mesenteric lymph nodes (MLNs) were smashed through 70-μm cell filter to prepare single-cell suspensions. In a 96-well plate, 1∼3 * 10^6^ lymphocytes/well were stimulated with PMA, ionomycin, brefeldin, and monensin for 4 h at 37°C under 5% CO_2_. The proportion of regulatory T cells was determined by flow cytometry.

### Detection of Concentration of 22 Amino Acids

The concentration of amino acids was detected following the method described by (Xu et al., 2016; Song et al., 2018). In brief, colonic tissues of about 40 μg were collected and homogenized in methanol (100 μl) on ice. Subsequently, 300 μl methanol and 280 μl water were added into the homogenate and mixed thoroughly. And other impurities were excluded by chloroform. The concentration of amino acids was determined by HPLC-MS/MS (Agilent 6420).

### Quantitative Real-Time Reverse Transcription Polymerase Chain Reaction (qRT-PCR)

The expression levels of gene pS6 and 4E-BP1 were detected *via* qRT-PCR. Total RNA of colonic tissues was isolated using RNA extraction kit (TaKaRa, 9767) and reversely transcribed into cDNA according to the manufacturer’s protocols. Sequences of the primers were as follows: Gapdh (forward 5′-CATCACTGCCACCCAGAAGACTG-3′ and reverse 5′-ATGCCAGTGAGCTTCCCGTTCAG-3′); pS6 (forward 5′-AGGTGGAACCTCCCTTTAAGCC-3′ and reverse 5′-CCAGAAAGACCTGGTTGGCACT-3′), and 4E-BP1 (forward 5′-GGAGAGCTGCACAGCATTCAGG-3′ and reverse 5′-GGAGGTATGTGCTGGTGTTCAC-3′).

### Statistical Analysis

Results in the current study are represented as mean ± SEM. One-way analysis of variance (ANOVA) was used to analyze the data. *P* < 0.05 was considered statistically significant.

## Results

### Berberine Attenuated DSS-Induced Colitis

**Figure 1:**
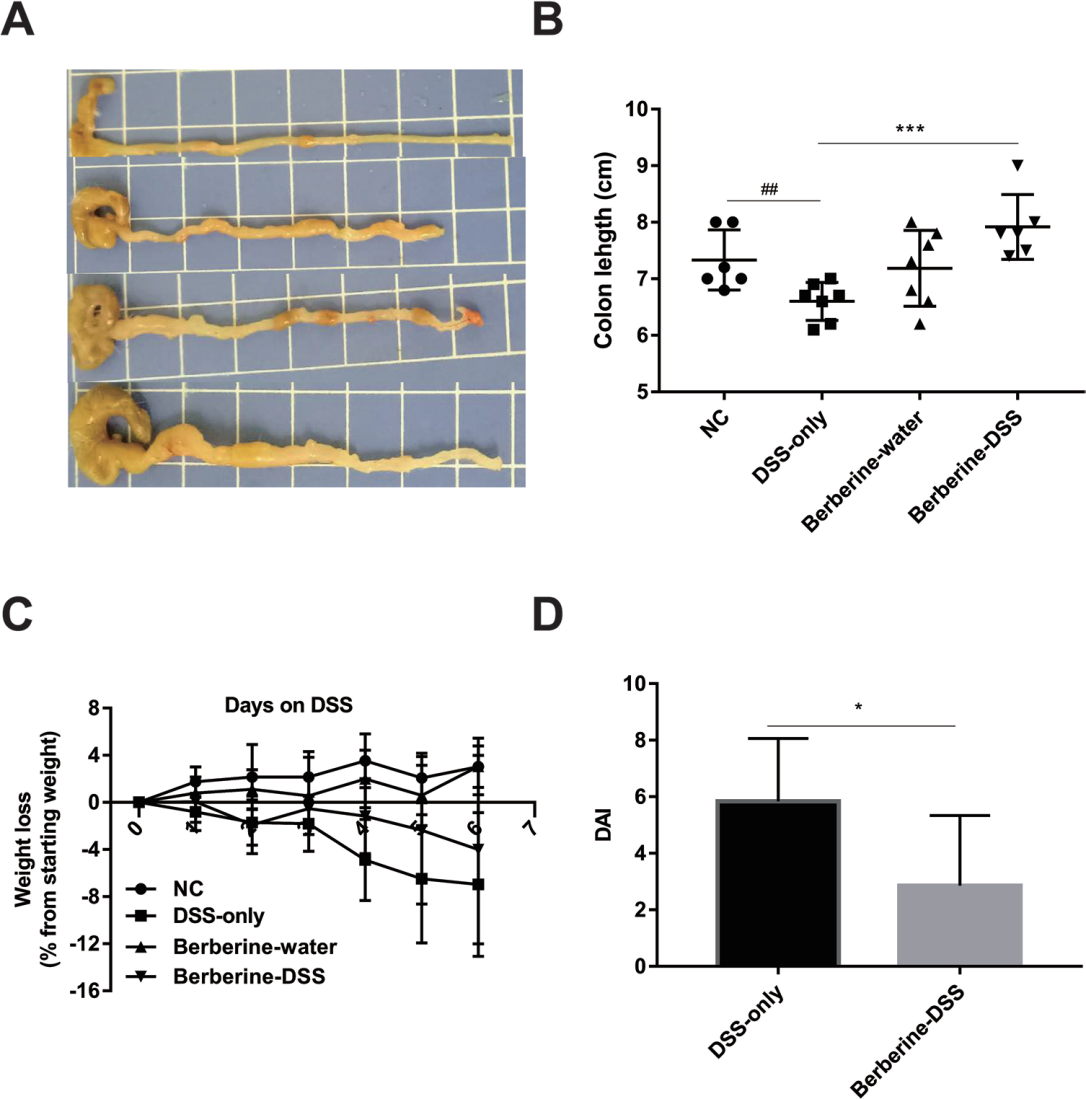
Berberine attenuated dextran sulfate sodium (DSS)-induced colitis. The pictures of colon **(A)**, colon length **(B)** and DAI **(D)** were determined on day 6 post DSS administration, and weight loss **(C)** was calculated daily. Data were pooled from two independent experiments with *n* = 7 mice per group. *P < 0.05, ***P < 0.001 and ^##^*P* < 0.01.

The clinical presentations of UC include fatigue, diarrhea, weight loss, and blood in stool. To evaluate the protective effect of berberine against acute colitis, the body weight was monitored every day, the colon length was measured, and the DAI was obtained by summing the scores of weight, rectal bleeding, and stool consistency. Mice in groups of normal control or berberine–water did not present symptoms of colitis during the experiment course. As shown in **Figure 1**, compared with the administration of DSS only, administration with berberine significantly alleviated symptoms of colitis indicated with significantly reduced DAI and longer colon.

### Berberine Decreased the Inflammation Condition in Mice With Colitis

**Figure 2:**
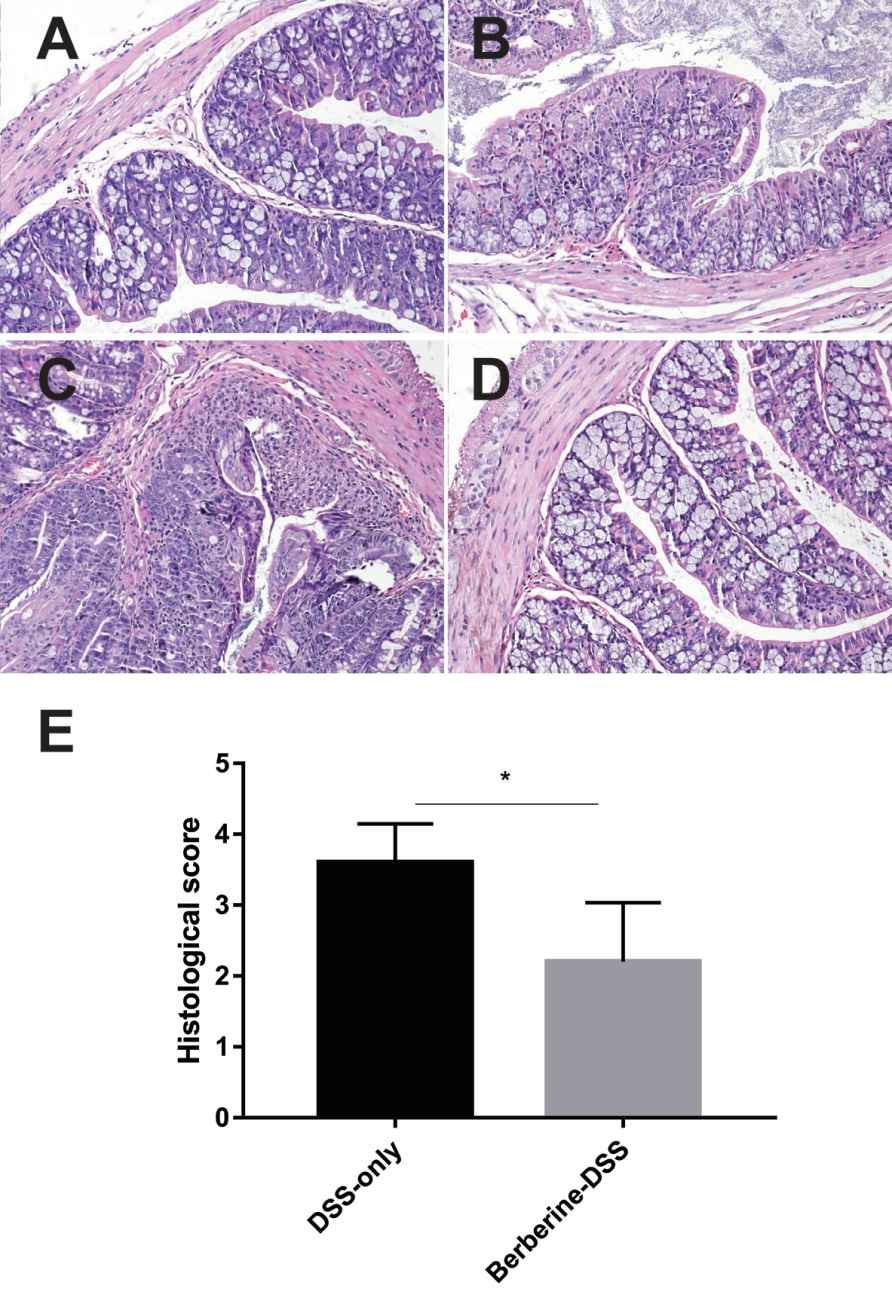
Effects of berberine on the histopathological changes in colonic tissues. Normal control **(A)**, DSS-only **(B)**, and administration with berberine (i.g., 50 mg/kg)–water **(C)** and berberine (i.g., 50 mg/kg)–DSS **(D)**, **(E)** Scores of the histopathological changes in colonic tissues (*n* = 7). *P < 0.05.

Ulcerations, destruction of crypt architecture and decreased crypt density, and depletion of goblet cells are features of inflammation in ulcerative colitis. The protective effect of berberine against colitis was evaluated by summing the scores of colonic tissue damage and the inflammatory cell infiltration in the colon. As shown in **Figure 2**, berberine decreased the severity of inflammatory condition in colonic tissue from mice with acute colitis compared with mice in the DSS-only group.

### Berberine Promoted the Differentiation of Treg Cells

**Figure 3:**
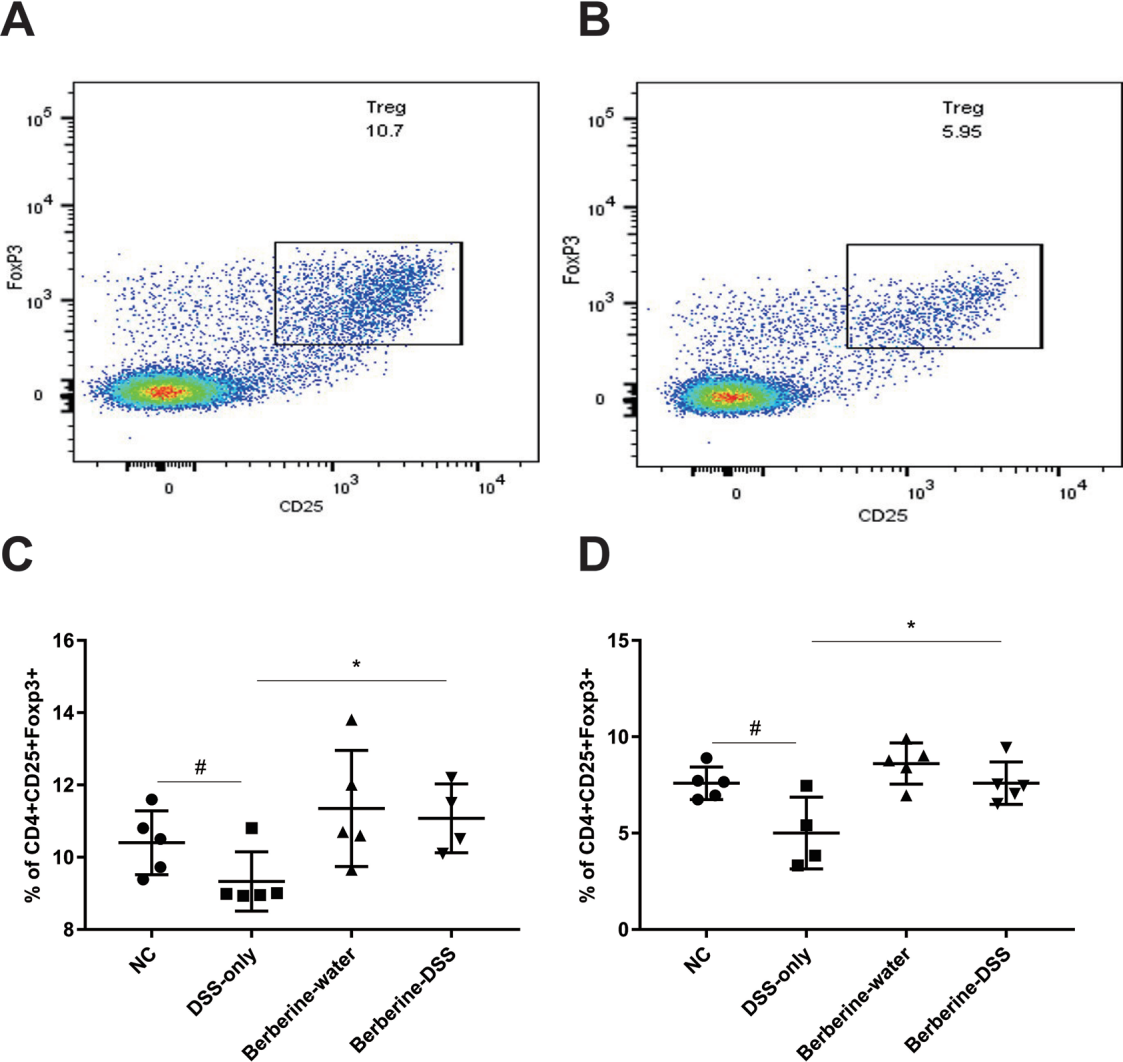
Effects of berberine on the differentiation. The proportion of regulatory T cells in mesenteric lymph nodes (MLNs) and spleens was evaluated by flow cytometry. **(A** and **C)** Tregs in MLNs (*n* = 7). **(B** and **D)** Tregs in spleens (*n* = 7). *P < 0.05, ^#^*P* < 0.05.

Treg cells are critical for the homeostasis of gut immune, and they are immunosuppressive and generally suppress or downregulate induction and proliferation of effector T cell such as Th1 and Th2. Moreover, Treg cells modulate the tolerance in intestine, and many studies have demonstrated the relationship between colitis and defects in development or function of Treg cells. To test if berberine could affect Treg cells in mice with colitis, lymphocytes from the spleens and MLNs were prepared and analyzed by flow cytometry. As shown in **Figure 3**, administration with berberine significantly increased the proportion of Treg cells, which suggested the anti-inflammatory activity of berberine.

### Changes in Concentrations of Amino Acids

**Figure 4:**
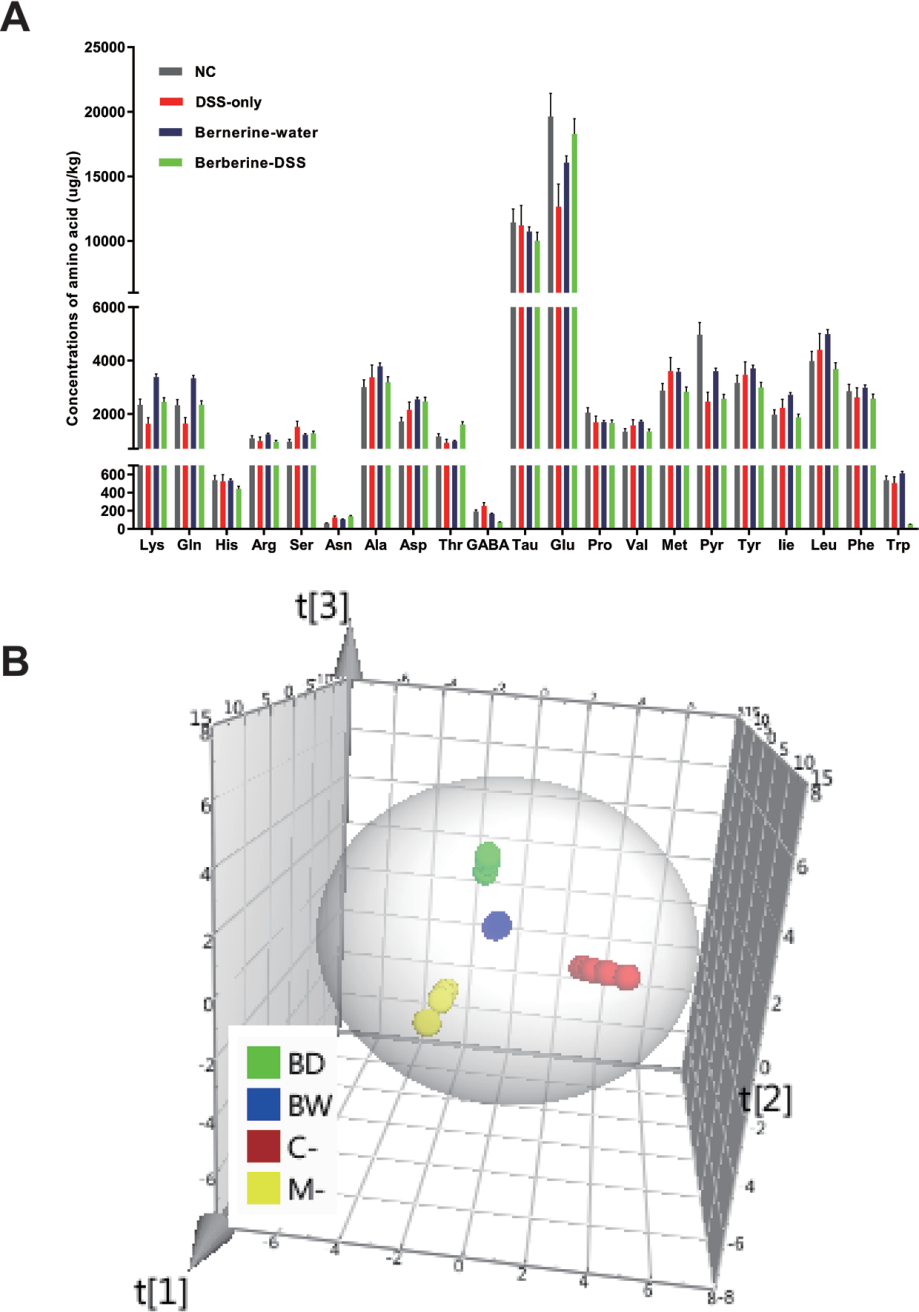
Changes in concentrations of amino acids. The concentrations of 22 amino acids were detected by HPLC-MS/MS **(A)**. Principal coordinates analysis (PCA) was used to analyze the changes of amino acids in SIMCA-P software **(B)**. Data were pooled from two independent experiments with *n* = 7 mice per group.

Targeted metabolomics was used to determine the concentrations of 22 amino acids related with various functions of host. Data obtained for the 22 amino acids were calculated by the Agilent MassHunter qualitative and quantitative analysis software and SPSS 16.0. It was found that concentrations of 10 amino acids including lysine, glutamine, serine, asparagine, aspartic acid, threonine, γ-aminobutyric acid, glutamic acid, valine, and isoleucine were significantly different between mice in normal control or mice administered with berberine, compared with mice fed with DSS only (**Figure 4A**). The concentrations of amino acids were imported to SIMCA-P software to further confirm their changes. Principal coordinates analysis (PCA) was used to perform unsupervised multivariate data analysis. As shown in **Figure 4B**, the four experimental groups in the current study were clearly discriminated from each other.

### Berberine Had an Effect on Glutamine and Glutamate Metabolism

**Figure 5:**
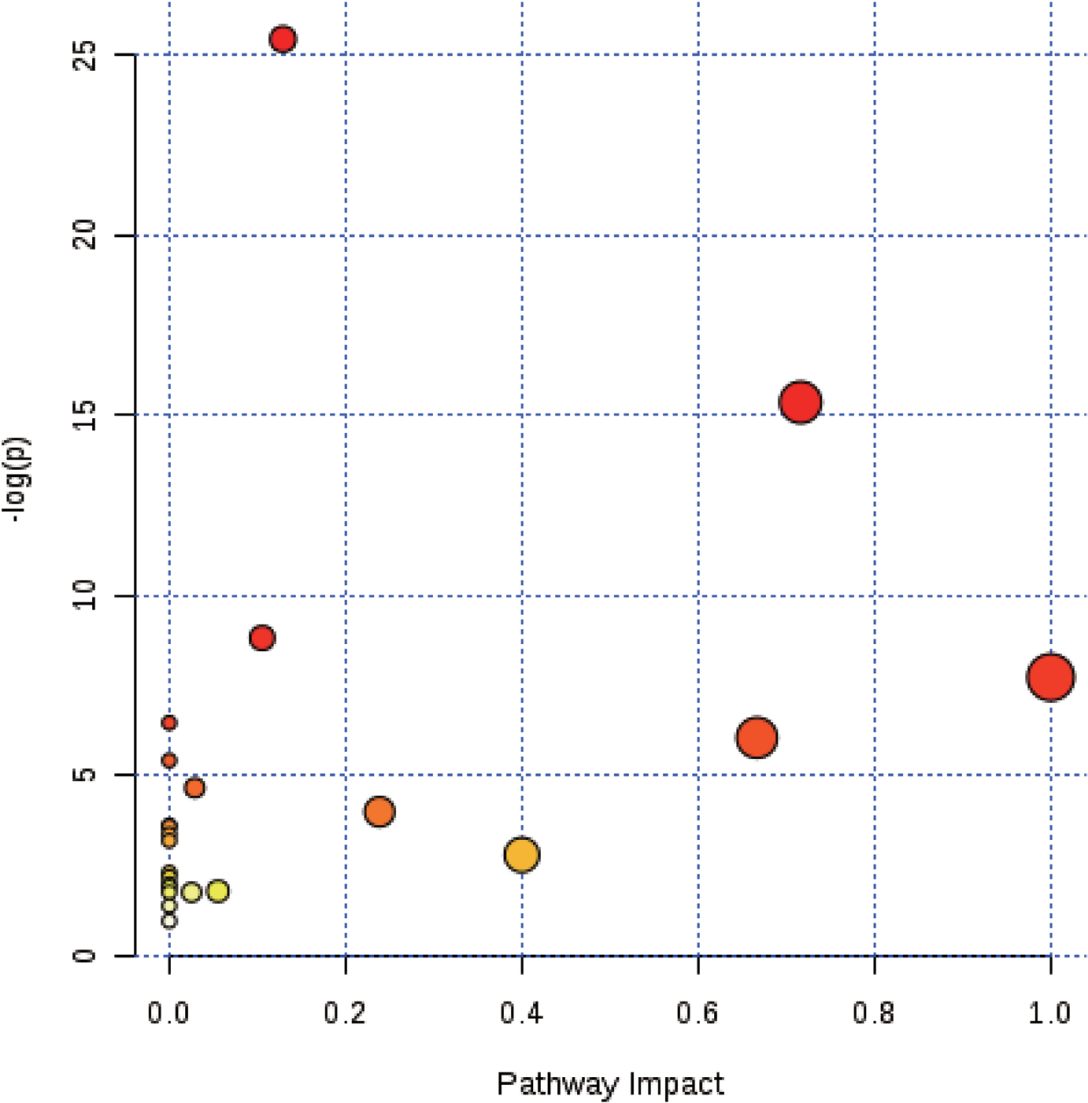
Berberine had an effect on glutamine and glutamate metabolism. MetaboAnalyst software was used to reveal the relevant pathway involved in the berberine’s regulation of immune response.

The functions of amino acids, which were significantly changed, were identified by human metabolome database, and the pathway analysis was determined by the MetaboAnalyst. It was found that the glutamine and glutamate metabolism were the most associated with the protective effect of berberine against colitis (**Figure 5**).

### Expression of Genes in mTORC1 Pathway

**Figure 6:**
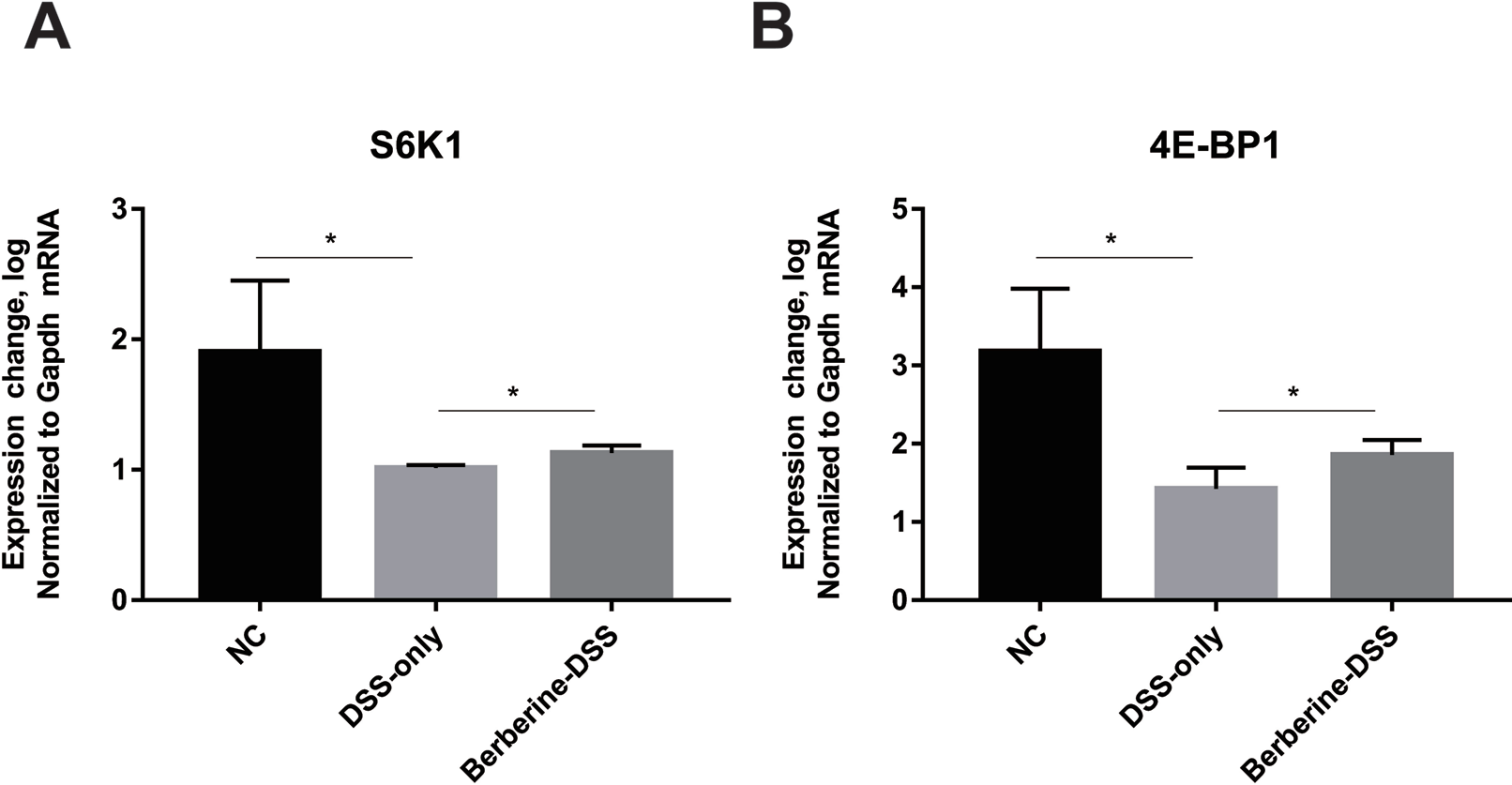
Effect of berberine on the mTORC1 pathway. The relative expression levels of gene S6K1 **(A)** and 4E-BP1 **(B)**. Data were pooled from two independent experiments with *n* = 7 mice per group. *P < 0.05.

It was reported that the metabolism of glutamine relates to mTORC1 pathway, which is related with the immune system of host. As a result, the current study assessed the expression levels of downstream effector genes of mTORC1 including S6K1 and 4EBP1. It can be found in **Figure 6** that administration with berberine could significantly increase the relative expression levels of S6K1 and 4EBP1.

### Berberine Had No Effect When mTORC1 Pathway Was Inhibited

**Figure 7:**
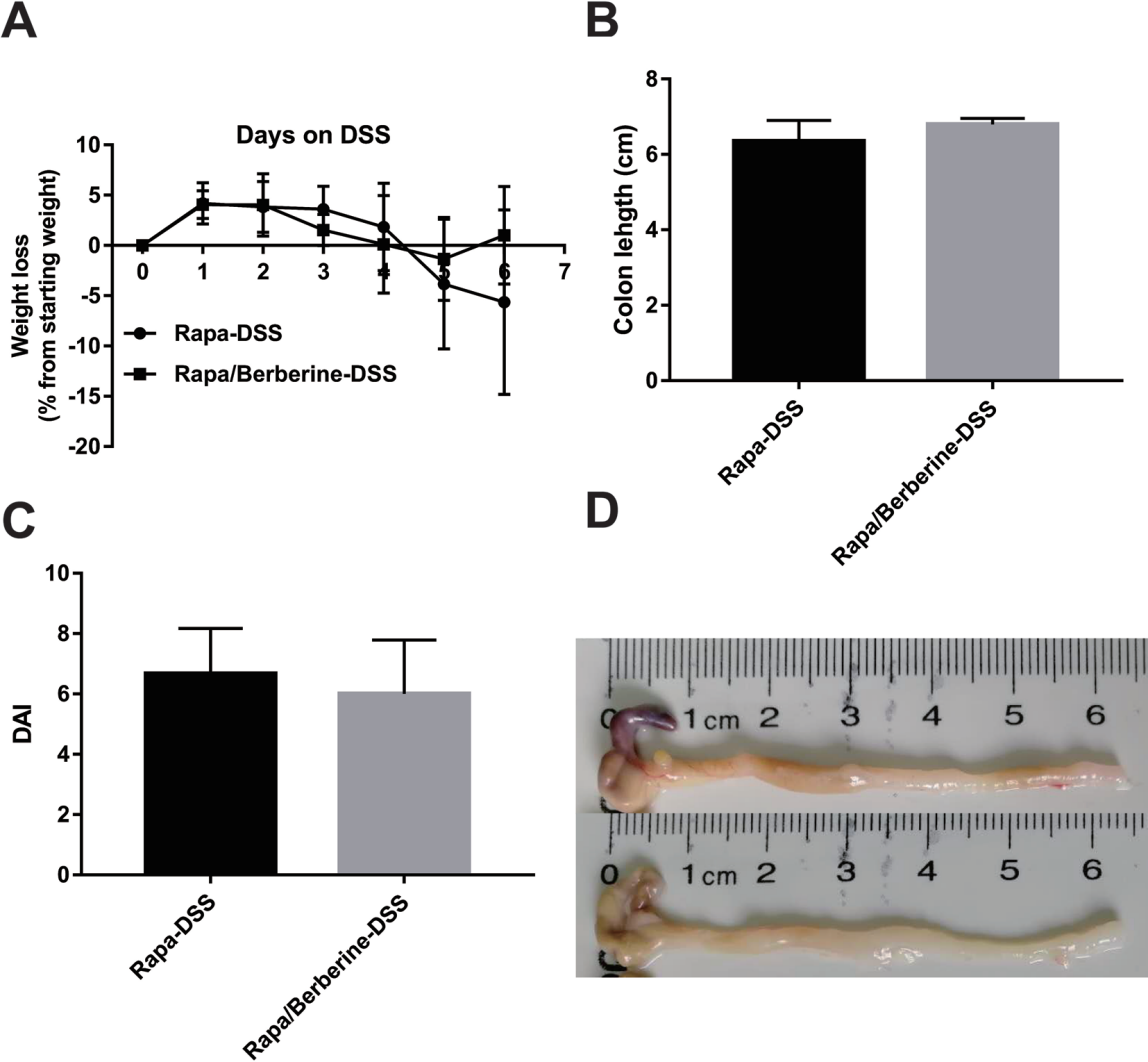
Effect of berberine against DSS-induced colitis when rapamycin was used to inhibit mTORC1 signaling. The weight loss **(A)** was calculated daily, and the colon length **(B)**, DAI **(C)** and pictures of colon **(D)** were determined on day 6 post DSS administration. Data were pooled from two independent experiments with *n* = 6 mice per group.

**Figure 8:**
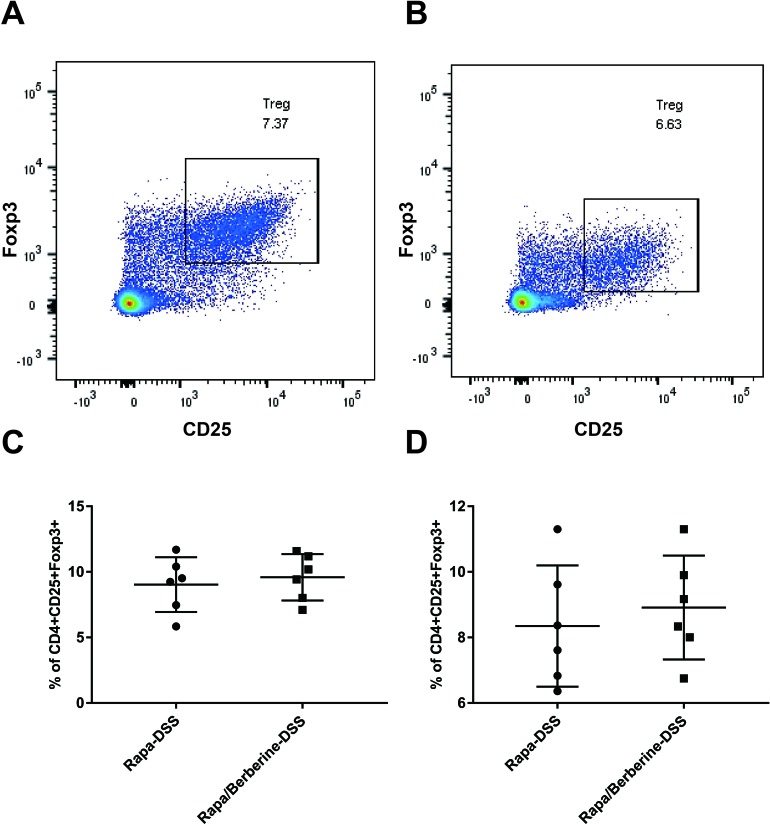
Effects of berberine on the proportion of Treg when rapamycin was used to inhibit mTORC1 signaling. The proportion of Treg in MLNs and spleens was evaluated by flow cytometry. **(A** and **C)** Tregs in MLNs (*n* = 6). **(B** and **D)** Tregs in spleens (*n* = 6).

Next, the effect of berberine was analyzed when mTORC1 was inhibited by rapamycin. As shown in **Figure 7**, the weight loss, colon length, and DAI were not different between the Rapa-DSS group and the Rapa/Berberine-DSS group. The proportion of Treg cells in MLNs and spleens was determined using flow cytometry as shown in **Figure 8**. Berberine did not have an effect on the proportion of Treg cells of mice in the Rapa/Berberine-DSS group (MLNs: 9.59 ± 1.77; spleens: 8.91 ± 1.58), compared with that of mice in the Rapa-DSS group (MLNs: 9.03 ± 2.09; spleens: 8.35 ± 1.85). These results together suggested that berberine had no effect while there was no mTORC1 activation.

## Discussion

Berberine is one kind of alkaloid component, and it has been demonstrated to possess various bioactivities such as anti-inflammatory, antitumor, and antioxidant properties (Dkhil et al., 2017; Almeer et al., 2018; Kim et al., 2018; Xu et al., 2018; Li et al., 2019). It has been reported that berberine could exert its protective effects against DSS colitis by improving intestinal barrier function and reducing inflammation and oxidative stress; and berberrubine, which has been reported to possess an anti-colitis effect similar to that of berberine with a much smaller dosage, is one of the metabolites of berberine (Yu et al., 2018). The mechanisms by which berberine exerts its activity have not been adequately investigated and need further research (Zhang et al., 2017). The possible mechanism of berberine may involve the blocking of the IL-6/STAT3/NF-κB signaling pathway (Zhu et al., 2019). It has not been reported before that mTORC1 pathway is involved in the anti-inflammatory activity of berberine.

The results of colon length and DAI were consistent with previous publications that suggest the therapeutic effect of berberine against DSS-induced colitis. Berberine has been reported to exert its anti-inflammatory activity by regulating immune system: V Li et al. revealed that berberine has an effect on adaptive immunity by inhibiting the differentiation of Th1 and Th17 cells (Li et al., 2015), and YH Li found that berberine could attenuate the relapse of colitis by suppressing Th17 responses (Li et al., 2016). In the current study, the proportion of Treg cells in the spleen and MLNs was analyzed. The result showed us that administration with berberine could significantly promote the differentiation of Treg cells.

The potential mechanism by which berberine could affect the Treg cells remains unclear. In our study, targeted metabolomics was applied to determine the concentrations of 22 amino acids. Concentrations of 10 amino acids, including lysine, glutamine, serine, asparagine, aspartic acid, threonine, γ-aminobutyric acid, glutamic acid, valine, and isoleucine, were found to change significantly after being treated with berberine. Then, the 10 amino acids were imported into MetaboAnalyst software to analyze the related pathway. The most relevant pathway was glutamine and glutamate metabolism.

The glutamine provides ketoglutarate-to-tricarboxylic acid cycle and is used as a source of nitrogen by proliferating mammalian cells (Csibi et al., 2013). Elevation of glutamine synthesis represents key adaptations to maintain amino acid balance, and it has been reported that glutamine reactivates mTORC1 specifically through its conversion to glutamate (Tan et al., 2017). And it has been reported that activation of mTORC1 pathway promotes the uptake and metabolism of glutamine, and cell proliferation (Csibi et al., 2014). The relative expression levels of S6K1 and 4EBP1, downstream effector genes of mTORC1, increased by treatment with berberine. The data were consistent with previous reports (Zeng and Chi, 2017).

In conclusion, berberine could protect mice against colitis by activating the mTORC1 pathway, which could promote the differentiation of Treg cells to ameliorate intestinal inflammation lesions.

## Ethics Statement

All the animal experiments protocols were reviewed and approved by the Animal Ethics Committee of the Shandong University of Traditional Chinese Medicine, and the animal experiments were conducted complying with the rules of the Shandong Administration Office of Laboratory Animals.

## Author Contributions

All authors including QL, XQ, XP, YS, LC, QX, LS, XW, HZ, DQ and ZW have made substantial, direct and intellectual contribution to the work and approved it for publication.

## Funding

This work was supported by the Key Research and Development program of Shandong Province (no. 2017CXGC1307 and no. 2018GSF119018) and Foundation of Shandong University of Traditional Chinese Medicine for Youth (no. 2018zk12).

## Conflict of Interest Statement

The authors declare that the research was conducted in the absence of any commercial or financial relationships that could be construed as a potential conflict of interest.
